# Design and Development of Inexpensive Paper-Based Chemosensors for Detection of Divalent Copper

**DOI:** 10.1007/s10895-023-03220-4

**Published:** 2023-04-10

**Authors:** Mithra Geetha, Kishor Kumar Sadasivuni, Maryam Al-Ejji, Nandagopal Sivadas, Bagmita Bhattacharyya, Farzana N. Musthafa, Sarya Alfarwati, Tamanna Jannat Promi, Sumayya Ali Ahmad, Sara Alabed, Dima Anwar Hijazi, Fatimatulzahraa Alsaedi, Faozia Nasser Al-Shaibah

**Affiliations:** 1 Center for Advanced Materials, Qatar University, P. O Box 2713, Doha, Qatar; 2 Central Laboratories Unit, Qatar University, P. O Box 2713, Doha, Qatar; 3 College of Pharmacy, Qatar University, P. O Box 2713, Doha, Qatar; 4 Biological and Environmental Sciences Department, Qatar University, Doha, Qatar

**Keywords:** Sensor, Monitoring, Dye system, Copper

## Abstract

**Abstract:**

Simple, portable, and low-cost paper-based sensors are alternative devices that have the potential to replace high-cost sensing technologies. The compatibility of the paper base biosensors for both chemical and biochemical accentuates its feasibility for application in clinical diagnosis, environmental monitoring, and food quality monitoring. High concentration of copper in blood serum and urine is associated with diseases like liver diseases, carcinomas, acute and chronic infections, rheumatoid arthritis, etc. Detection of copper concentration can give an early sign of Alzheimer disease. Apart from that genetic Wilson's disease can be detected by evaluating the concentration of copper in the urine. In view of the above advantages, a novel and the highly sensitive paper-based sensor has been designed for the selective detection of Cu^2+^ ions. The fast and highly sensitive chemiresistive multi-dye system sensor can detect Cu^2+^ ions selectively in as low as 2.23 ppm concentration. Least interference has been observed for counter ion in the detection of Cu^2+^. Copper chloride, nitrate, and acetate were used to validate the detection process. This assay provides a very high selectivity of Cu^2+^ ion over other metal cations such as Na^+^, Mg^2+^, Ca^2+^, etc. The easy preparation and high stability of dye solutions, easy functionalization of the paper-based sensors, high selectivity over other cations, low interference of counter anion, and significantly low detection limit of 2.23 ppm make it an effective Cu^2+^ ion sensor for real-time application in near future.

**Graphical Abstract:**

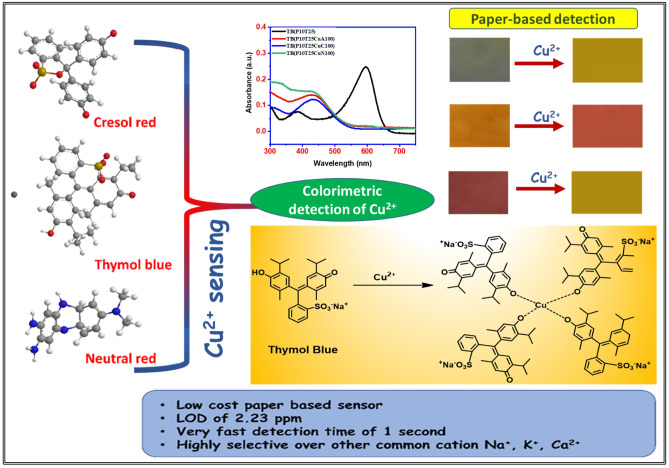

## Introduction

Copper is a vital trace element for the human body for the proper functioning of the organs and keeping good health. Starting from tissue development to blood formation copper-based metalloenzymes play an essential role. Despite being so important for human health, excess copper is very harmful and can cause neurodegenerative diseases like Parkinson's, Alzheimer's, and Wilson's disease, gastrointestinal disturbance, and even can cause serious damage to the liver and kidney [1–6]. Overconsumption of Cu (II) may cause blood pressure and respiration rate to increase. [7, 8]. In addition, it has been found that diseases and Cu (II) metabolism issues are closely related [9]. The biggest challenge in this area is developing simple, very accurate, and delicate tools and systems to safeguard the public from heavy metal poisoning. As a result, there is a strong need for quick, simple, and reliable copper detection techniques. The National Research Council recommends a daily copper consumption of 0.4–0.6 mg for infants, 1.5–2.5 mg for children, and1.5–3.0 mg for adults [10, 11]. In addition, the U.S. Environmental Protection Agency (EPA) established that the Cu^2+^ ion limit in drinking water is 1.3 ppm [12]. It mostly manifests in water as a divalent ion that forms complexes with ligands that contain halogens, oxygen, and sulfur [13–15]. Organisms are primarily exposed to copper through the intake, inhalation, and dermal contact [16, 17]. It is imperative to take action to monitor heavy metal discharge into the environment due to the damaging consequences of heavy metal pollution.

Drinking water might be a potential source of Cu (II) condensation because it is produced or used in industry 18. Large amounts of copper in drinking water are harmful to aquatic plants and animals, including people, because the cell membranes of aquatic plants prevent elements from passing through the cell walls [18–21]. Currently, in the last few years, tremendous efforts have been put forward for the detection of copper in aquatic and biological systems because of its possible toxic effect on human health and the ecosystem. The high solubility of copper ions in water imparts more tread to human health by imparting in the food chain. Cu^2+^ ions have been detected thus far using a range of technologies, including atomic absorption/emission spectroscopy (AAS/AES), surface plasmon resonance (SPR), high-performance liquid chromatography (HPLC), and inductively coupled plasma mass spectrometry (ICP-MS) [22–27]. These techniques have significantly improved the ability to detect Cu^2+^ ions in various sample types. However, these conventional techniques frequently call very pricey apparatus, intricate sample preparation, and qualified operators.

The selectivity of the Cu^2+^ is of prime importance while preparing any sensor or analytic techniques because in many cases interference from similar cations such as Ni^2+^, Hg^2+^, and Co^2+^ are overserved in copper-based sensors. Therefore, low-cost, effective, and selective detection of Cu^2+^ is highly desirable. Colorimetric detection of ions is another immerging technique developed in recent times, which required no sophisticated analytic instrument, and detection can be observed even by the naked eye, by the change in the color. For example, Joo et al. developed a chemosensor with a detection limit of 0.14 M by combining N-aminophthalimide and 8-hydroxyjulolidine-9-carboxaldehyde in a buffer/DMF solution (3/2, v/v, 10 mM bis–tris, pH7.0) [28]. Jin and Han developed CdSe/ZnS quantum dots (QDs) modified with hexadecyl trimethylammoniumbromide (CTAB), enabling very sensitive and focused fluorometric detection of Cu^2+^ ions in the presence of thiosulfate [29]. The detection of trace levels of Cu^2+^ ions in aquatic habitats and living systems, however, continues to be difficult. Therefore, the creation of a practical, quick, and sensitive sensor for Cu^2+^ detection in the environment and living systems is urgently needed.

Metal-selective sensing has been of great interest for applications ranging from environmental assays to industrial quality control, but sensitive metal detection for field-based assays has been elusive. The novelty of this work is for the detection of copper ions. The importance of copper ion is evidenced by the following statements along with the references. Several biological processes, such as signaling, metabolism, and catalysis, depend on transition metals [30, 31]. Copper, an important transition metal are known to be essential to biology; physiological imbalances in this metal can cause a variety of different health issues [32, 33]. Copper is an essential cofactor for about 30 enzymes and aids in the generation of ATP, catecholamine biosynthesis, and the defense of the cell against oxygen free radicals, among other biological activities [34, 35]. In physiological settings, copper is present as both Cu(I) and Cu(II), although Cu(II) is more stable and highly redox active, making it useful as an antioxidant [36]. Nevertheless, alterations in copper (II) homeostasis have been associated with the emergence of neurodegenerative illnesses like Alzheimer's, Parkinson's, Menkes, and amyotrophic lateral sclerosis and can be extremely toxic to cells (ALS). Along with contributing to disease, copper (II) can be an unwelcome pollutant in soils, water, and even jet fuel. Low quantities can have harmful effects; for instance, sub-micromolar concentrations of copper can hasten the decomposition of fuels and have an impact on aquatic microbes [37, 38].

Paper-based colorimetric device is a recently developed disposable device that attained remarkable success in the least time. High reaction rates, minimal sample consumption, and excellent biocompatibility are a few of the desired parameters in the development of any sensor. Paper-based devices are practical for everyday usage because they are inexpensive and simple to operate. Still there is a long way to achieve highly sensitive, selective, and stable sensors for commercial application purposes. We, therefore, developed an alternative method in this study that would efficiently and selectively detect Cu^2+^ ions while also enabling copper detection and readout of equivalent quantities both directly from an aqueous solution and in a colorimetric analysis. For the detection of Cu^2+^ ions in an aqueous solution, even more, practical paper sensors have also been produced. This study demonstrates the possibility of real-time, extremely sensitive, probe-free visual detection of Cu^2+^ ions in the environment and living systems using colorimetry.

## Experiments and Method

### Materials and Instruments

All the chemicals required during the experiments, copper(II)chloride.2H_2_O, copper(II)acetate, copper(II)chloride, Thymol blue, Cresol red, and Neutral red (0.1%) were purchased from Sigma Aldrich and used as received without prior treatment. Millipore Milli-Q water system supplied purified water was used for carrying out all the experiments. Analysis of the colorimetric results was done by using Biochrom UV spectroscopy from China to perform the UV–Vis spectroscopy characterization using a 190–1100 nm scanning range. The dye samples were examined in a 250–750 nm scanning range at a medium scan speed. Characterization involved a step input of 1 nm and a bandwidth of 2 nm.

### Methods

#### Preparation of the Copper Solutions, Dyes, and Characterization

Thymol blue, Cresol red, and Neutral red dye solutions were prepared by dissolving 0.1 g of dye in 100 mL of DDI water. After the solution was prepared, a dilution of 1:100 dye to distilled water was used as the standard for further experiments. Finally, 3 mL of these solutions was taken into 45 vials, 15 for each dye, divided into 9 rows. For each dye solution pH was adjusted to acidic (3 and 5), neutral, and basic (10 and 12) pH. After that, 100 ppm copper nitrate, copper chloride, and copper acetate solutions (10 mg in 100 mL DW) were prepared. Then, 1 mL of each prepared copper solution was added to a row of dye. A change of color and time was observed for all the dye solutions. UV spectrophotometer was used to perform to investigate the efficiency of dye for the detection of Cu^2+^ at room temperature. A graph between absorbance and wavelength was drawn after the characterization and data evaluation. The paper sensor was imaged using a Samsung SCX-3400 scanner and ImageJ open-source software.

#### Optimization of Kinetic Parameters

##### Effect of pH on Detection Efficiency

The dye solution’s pH was adjusted to acidic (3 and 5), neutral, and basic (10 and 12) pH for comprehending pH's impact. Now, 1 mL of prepared copper stock solution was added to each of the dye solutions. A change in color and time was observed for all the dye solutions and UV–visible spectroscopy was used as a tool to evaluate the effect of pH and detection of Cu^2+^ ion by the dye solution at different pH.

##### Effect of Temperature on Cu^2+^ Detection

To investigate the effect of temperature on the detection efficiency of the dye, dye solutions were heated to temperatures 25, 40, 60, 75, and 90 ℃. Before adding the copper salt solution, the stability of the dye solution was ensured by keeping it at a certain temperature for 5 h. After that copper stock solution was added to the dye solution and the changes observed were confirmed by UV–visible spectroscopy.

##### Effect of Concentration

To determine the effect of concentration on the detection efficiency different concentrations of copper solution ranging from 5–100 ppm were added to the dye solution and their changes were investigated with a UV–visible spectrophotometer.

##### Selectivity of Cu^2+^ Over Other Cation

For the selectivity analysis, 1 mL of 100 ppm sodium hydroxide, magnesium sulfate, potassium chloride, sodium chloride, and calcium carbonate were added to the dye solutions at room temperature and the color change was observed. To determine the concentration of the test solution, the temperature of the dye biomarker mixture, pH, and the selectivity of the dye solutions, the difference in absorbance was noted.

### Functionalization of Whatman Paper for Cu^2+^ Detection

An irreversible calorimetric method was applied for the detection of copper ions in the solution. For that purpose, Whatman filter paper was covered with one of the dyes Cresol red, Thymol blue, and Neutral red at a concentration of 0.003 M at room temperature. The dye solution was allowed to stand for 1 h before being transferred to a petri dish at pH 10. The Whatman paper was submerged in the dye solution for 1 h and dried in air at room temperature overnight.

### Image Processing by ImageJ 1.47 Software

The pictures of the paper sensor were collected using a scanner Samsung SCX-3400 with 300 dpi resolution to observe the changes in colour change after and before the addition of copper solution at different concentrations with various dyes. Pictures were saved in JPEG format and processed in RGB format using the Image J software, an open-source software [39]. To filter out the colors which are not associated with the colored complex to be discovered during analysis, the color threshold was adjusted for all images. The “color threshold” can be accessible for examination by selecting “Image” from the ImageJ menu! “Adjust”! “Color Threshold,” as it's known. Saturation, hue, and brightness can be adjusted using the HSB bottom present in this window.

The hue was changed in such a way that only the color of interest is visible. For each analysis, threshold ranges were established. The images were then overturned ("Edit" "Invert") and converted to an 8-bit grayscale ("Image" "Type" "8-bit"). For each RGB channel (red, blue, and green, "Image" "Color" "Merge Channel"), the Mean Gray Value (MGV) was calculated by first selecting "mean gray value" and "limit to threshold" in the "Set measurements window," which can be retrieved from the ImageJ menu by selecting "Analyze" "Set measurements." The wand tool was used to choose each region since it automatically located the edge of an item and traced its form. By selecting "Analyze" and "Measure," the gray intensity of the designated region was measured. The RGB channel was then chosen. After that, the data was imported into Microsoft Excel 2019 to generate the various calibration curves for each concentration. IUPAC regulations calculated the colorimetric detection limits using 3SB/S, where SB and S stand for standard deviation and slope, respectively [40, 41].

## Results and Discussion

To construct a highly sensitive colorimetric volatile biomarker, the Copper Nitrate, Copper Acetate, and Copper Chloride, concentrations varying from 5 to 100 ppm were added to acidic, basic, and neural medium of Cresol red, Thymol blue, and Neutral red dye and observed for any visible color change. In addition, the response time, pH effect, temperature effect, concentration effect, and selective nature of the dyes were studied and analyzed. Table 1 explains the list of notations that are used. Here, P stands for pH, x for a particular pH, T for temperature, and y for a particular temperature. Z represents the biomarker's concentration.

**Table 1:** List of notations used

Dye	Dye Indication	Specific pH (Px), specific temperature (Ty in °C), and biomarker [Cu(NO_3_)_2_/Cu(CH_3_COO)_2_/CuCl_2_] − concentration (z in ppm) indication
Thymol Blue	TB	$\mathrm{TB}({\mathrm{P}}_{\mathrm{x}}{\mathrm{T}}_{\mathrm{y}}{\mathrm{A}}_{\mathrm{z}})$
Cresol Red	CR	$\mathrm{CR}({\mathrm{P}}_{\mathrm{x}}{\mathrm{T}}_{\mathrm{y}}{\mathrm{A}}_{\mathrm{z}})$
Neutral Red	NR	$\mathrm{NR}({\mathrm{P}}_{\mathrm{x}}{\mathrm{T}}_{\mathrm{y}}{\mathrm{A}}_{\mathrm{z}})$
Cu(NO_3_)_2=_ CuN; Cu(CH_3_COO)_2=_ CuA; CuCl_2_ = CuC		

### Response Time and pH Effect

For the assay, 1 mL of the biomarker (CuA/CuN/CuC) solution along with 5 – 100 ppm (In CR, TB, and NR dyes) concentrations was added to the dye solutions with the pH values of 3, 5, 7, 10, and 12 at room temperature. By estimating how long it took between adding the biomarker and noticing the associated visible color change in the dye solution, the dye response times were estimated. Thymol blue, Cresol red, and Neutral red successfully detected copper (Table 2).

**Table 2:** Calorimetric detection of Cu^2+^ using different dyes

Sl. No	Dye solution + Copper compound	Average response time (s)
1	TB(P10T25CuA)	1
2	TB(P10T25CuN)	
3	TB(P10T25CuC)	
4	CR(P10T25CuA)	
5	CR(P10T25CuN)	
6	CR(P10T25CuC)	
7	NR(P10T25CuA)	
8	NR(P10T25CuN)	
9	NR(P10T25CuC)	

**Fig. 1:**
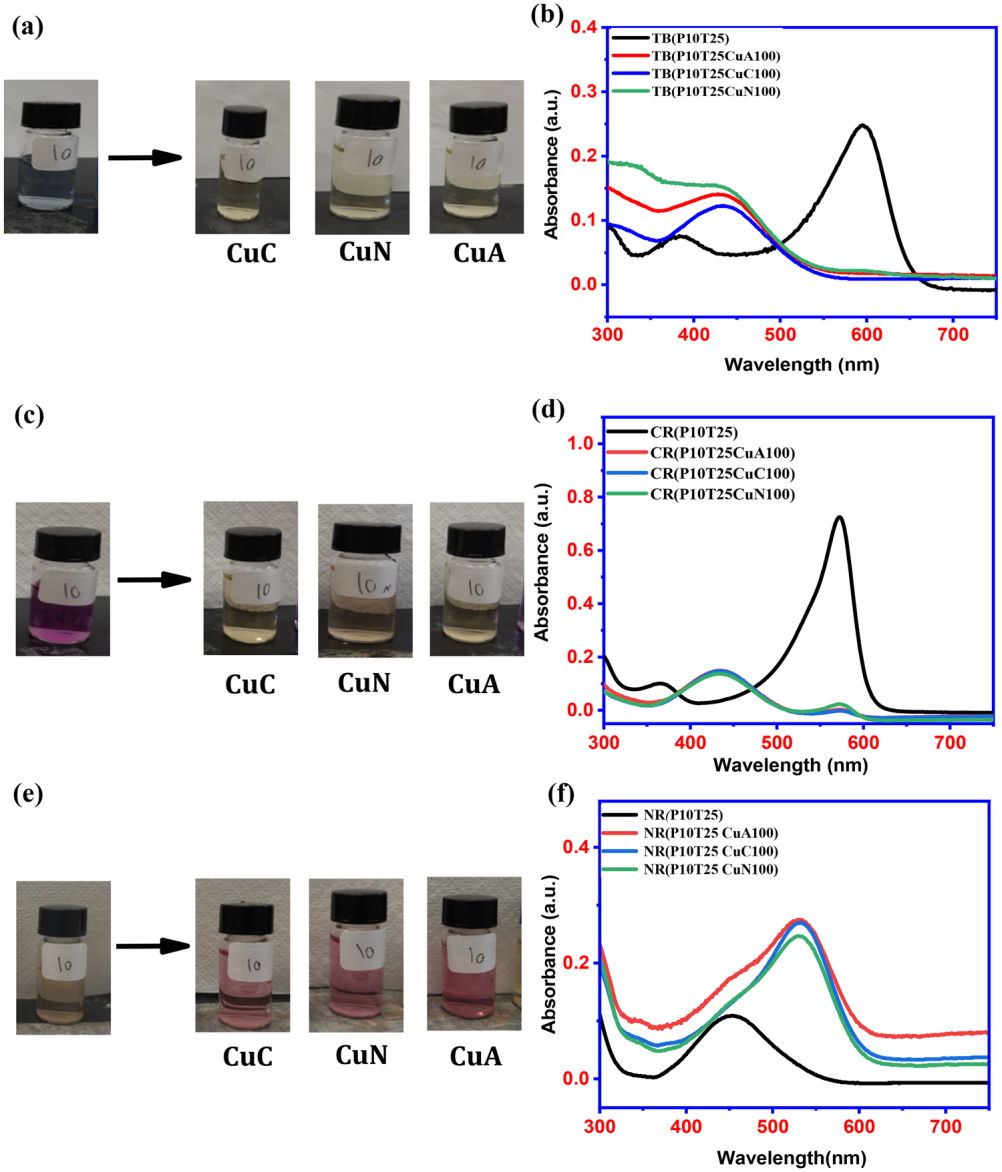
Copper ion detection using various pH-adjusted dyes at ambient temperature. **a** before and after addition of CuCl_2_, Cu(NO_3_)_2_, Cu(OAc)_2_ to Thymol blue dye at pH10; **b** corresponding UV–vis spectra of before and after addition of 100 ppm CuCl_2_, Cu(NO_3_)_2_, Cu(OAc)_2_ to Thymol blue **c** before and after addition of CuCl_2_, Cu(NO_3_)_2_, Cu(OAc)_2_ to Cresol red dye at pH10; **d** corresponding UV–vis spectra of before and after addition 100 ppm CuCl_2_, Cu(NO_3_)_2_, Cu(OAc)_2_ to Cresol red **e** before and after addition of CuCl_2_, Cu(NO_3_)_2_, Cu(OAc)_2_ to Neutral red dye at pH10; **f** corresponding UV–vis spectra of before and after addition 100 ppm CuCl_2_, Cu(NO_3_)_2_, Cu(OAc)_2_ to Neutral red

Figure 1 a shows the color of the Thymol blue dye solution at pH 10 before the addition of copper(II) solutions. Change in the Thymol blue dye solution after adding 100 ppm of copper solutions were shown in Fig. 1a. From Fig. 1a, it can be inferred that the pH 10 dye solution color tends to diminish with a copper solution. It can be concluded that the color reaction of Thymol blue and copper solution can be completed instantly within a second for copper solution concentration as low as 75 ppm and occurred in the pH range of 10. The emergence of a new absorption band centered at ~ 434.2 nm with a.u. ~ 0.123 for copper chloride, a.u. ~ 0.141 for copper acetate, and 0.153 for copper nitrate from 596.5 nm with a.u. ~ 0.247 was observed in the UV–vis absorbance spectra (Fig. 1b).

For the copper solution assay in the Cresol red dye solution, a visible color change was noticed in the neutral solution for all copper solutions concentrations that is, 75 – 100 ppm. Figure 1c shows the color change in the Cresol red solution after the addition of 100 ppm copper solutions. An apparent visible color change from purple to light yellow was observed in the dye solution of pH10. The response time of the pH10 solution is instant. The peak in the UV analysis (Fig. 1d) exhibited a shift from 571.3 nm with a.u. ~ 0.73 to 433.3 nm with a.u. ~ 0.15 for all copper solutions. Figure 1e shows the color change in the Neutral red dye after adding 100 ppm copper solution.

An apparent visible color change from light yellow to light pink is observed in the dye solution of pH10, along with a change in intensity from a.u. of 0.109 to 0.25 for copper nitrate and 0.27 for copper acetate and chloride (Fig. 1f). And a change from light to darker pink in the dye solution of pH7, along with a change in intensity from a.u. of 0.085 to 0.246 for copper acetate and 0.273 for copper nitrate and chloride (Fig. 1f). The observable color change can be distinguished at a concentration of copper solution as low as 100 ppm, offering a convenient approach to detecting copper by the unaided eye.

**Fig. 2:**
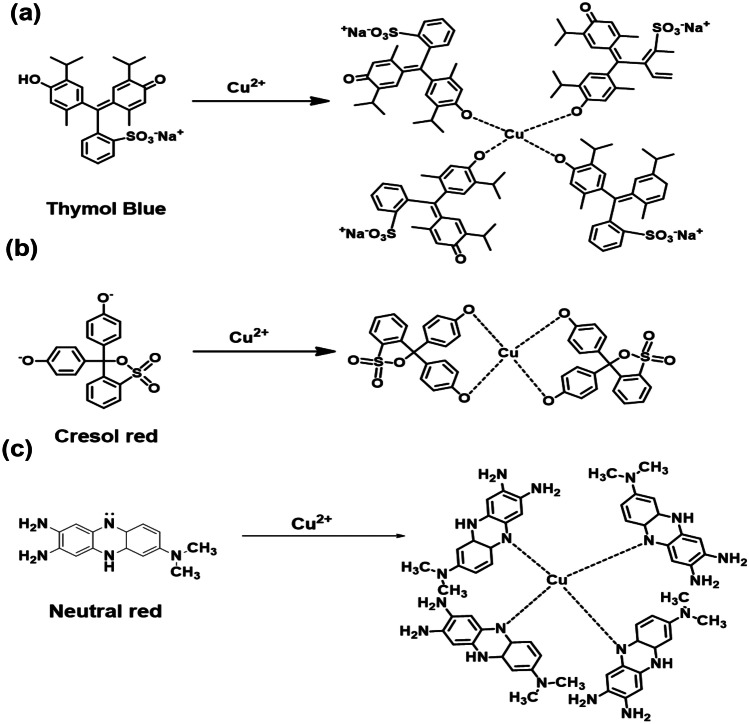
Possible mechanism of action of Cu(II) with **a** Thymol blue, **b** Cresol red, and **c** Neutral red dye solutions at pH 10

An example of the reaction of dyes with the copper solution is given below. In the strong basic medium, both Thymol blue and Cresol red dye will present in anionic form by releasing its protons from the hydroxyl groups. Thus easy coordination with the Cu(II) ion is possible at the high pH of 10. The plausible reaction involved in the interaction of Thymol blue and Cresol red is demonstrated in Figs. 2a, and b. Other than that in the case of Neutral red, no free hydroxyl groups are present. But at high pH of 10, the -NH group of the mid-six membered ring can release a proton, and that can be stabilized by conjugation in the cyclic system. When it gets in contact with the Cu(II) ion it can interact immediately and can stabilize copper in solution. This shows a color change from red to yellow in the pH range of 10.

### Effect of Temperature

Inertness to change in the surrounding physical environment is of utmost importance to ensure its stability and reliability in a commercial application. The stability of dye systems and their physical property can be altered at the molecular level by variations in temperature. The rise in vapor pressure attributed to the increase in temperature can affect the sensing ability, sensitivity, and also response time of the sensors. For a sensor to have potential in the commercial application must have the least impact on response time with temperature variation. Thus, to investigate the effect of copper ion detection ability all three dye solutions were heated to 25, 45, 60, 75, and 90 ℃ before adding the ion solution. To ensure the stability of the dye in different temperature ranges solutions were kept at a certain temperature for 1 h before adding the copper solutions. This temperature-dependent study was carried out at pH 10 owing to its uniform detection possibility with all the dyes.

**Fig. 3:**
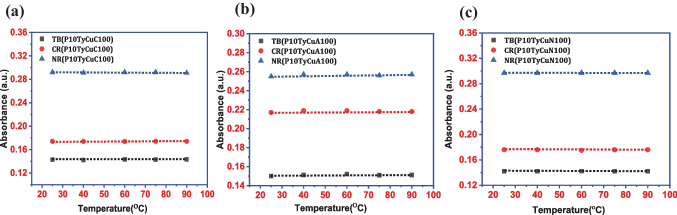
Temperature effect of dye solutions at 25, 40, 60, 75 and 90 °C with **a** copper chloride, **b** copper acetate and **c** copper nitrate pH 10

For the detection of copper all the prepared copper solutions namely copper(II)acetate, copper(II)chloride, and copper(II)nitrate were used. Regardless of temperature changes, the absorption intensity remained nearly consistent for all dye solutions (Fig. 3). Analysis showed that Thymol blue has an absorbance variation of 0.142 ± 0.001 in copper nitrate and 0.15 ± 0.001 in copper acetate and chloride, whereas, for Cresol red, the variation was from 0.219 ± 0.001 in copper acetate and 0.176 ± 0.002 in copper nitrate and chloride. Similarly, Neutral red exhibited a change in absorbance close to 0.257 ± 0.001 in copper acetate and 0.295 ± 0.003 in copper chloride and nitrate. Similar procedure was adopted for effective and selective detection of dye-based acetone [42], ethanol, methanol, and formic acid [43] previously, and no effect of temperature on the absorption efficiency has been observed in that case also.

### Sensitivity Analysis and the LOD of Dye Solutions

The dye solutions Cresol red, Thymol blue, and Neutral red showed very good activity for the detection of the copper ion with different counter ions like acetate, nitrate, and chloride. This also demonstrated the effectiveness of the sensitivity of the dye solution towards copper detection in presence of different counter ions. Thus, after successful detection of the copper ion with different counter ion in the pH range of 10, the sensitivity of the dye solutions were further investigated with different concentration of copper solutions ranging from 5–100 ppm. The detection of copper ions for all copper acetate, chloride, and nitrate solutions has been observed even at a low concentration of 5 ppm. A shift in the absorption maxima has been observed for each dye solution with all the copper solutions. Considering the linear relationship between absorption and concertation stated by Beer-Lamberts las, concentration vs absorbance graphs were plotted for all the dye solutions in presence of all the copper salt solutions using a linear calibration curve.

**Fig. 4:**
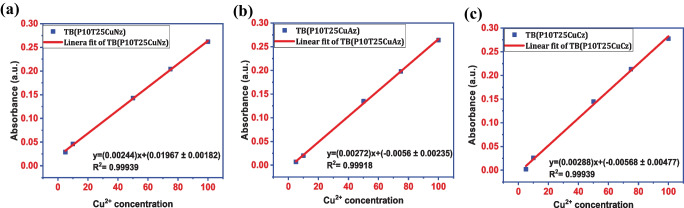
**a** Copper acetate calibration plot; **b** Copper nitrate calibration plot and **c** Copper chloride calibration plot for Thymol blue

A linear fitting was done to estimate the LOD using the 3σ/m criterion, where m is the slope of the calibration plot and σ is the standard deviation of the intercept. The calibration curve was plotted by considering the dye's peak absorbance. Figure 4a − c shows the calibration curve for Thymol blue dye solution in presence of copper nitrate, copper acetate, and copper chloride solution in different concentrations. The estimated LOD for the detection of copper acetate is found to 2.59 ppm [y = (0.00272)x + (-0.0056 ± 0.00235)]; R^2^ = 0.99918]. A detection limit of 2.23 ppm was obtained while using nitrate as a counter ion with copper(II) ion [y = (0.00244)x + (0.01967 ± 0.00182)]; R^2^ = 0.99939]. Similarly with copper chloride solution LOD is found to be 4.9 ppm [y = (0.00288)x + (-0.00568 ± 0.00477)]; R^2^ = 0.99939].

**Fig. 5:**
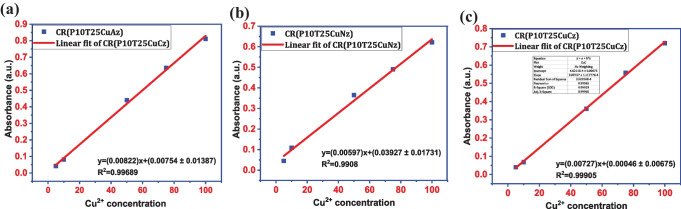
**a** Copper acetate calibration plot; **b** Copper nitrate calibration plot and **c** Copper chloride calibration plot for Cresol red

Figure 5a − c shows the calibration curve for Cresol red dye solution in presence of copper nitrate, copper acetate, and copper chloride solution in different concentrations. The LOD of Cresol red In presence of copper acetate was calculated to be 5.06 ppm [y = (0.00822)x + (0.00754 ± 0.01387)]; R^2^ = 0.99689]. Data obtained from the calibration plot of Cresol red in presence of copper nitrate showed a LOD of 8.6 ppm, according to the linear fit data [y = (0.00597)x + (0.03927 ± 0.01731)]; R^2^ = 0.9908]. Similarly, the calibration curve in presence of copper chloride was plotted from 5–100 ppm, and a lower detection limit of 2.78 ppm was observed for copper sensing, according to the linear fit data [y = (0.00727)x + (0.00046 ± 0.00675)]; R^2^ = 0.99905].

**Fig. 6:**
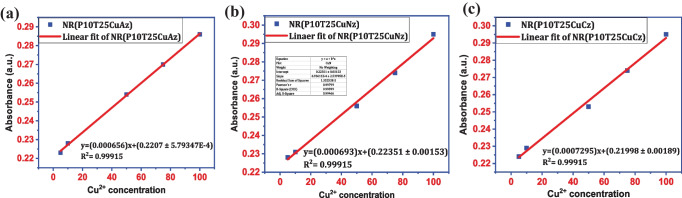
**a** Copper acetate calibration plot; **b** Copper nitrate calibration plot and **c** Copper chloride calibration plot for neutral red

Figure 6a-c shows the calibration curve for Neutral red in presence of copper acetate, copper nitrate, and copper chloride respectively. The estimated LOD of 2.64 ppm was obtained for the detection of copper acetate [y = (0.000656)x + (0.2207 ± 5.79347E-4)]; R^2^ = 0.99915]. Like copper acetate, the calibration curve of Neutral red was plotted in presence of copper nitrate. The linear fit to the data revealed a LOD of 6.61 ppm toward copper ion sensing by the Neutral red dye [y = (0.000693)x + (0.22351 ± 0.00153]; R^2^ = 0.99915]. Similarly, the linear fitting was performed in the range of 5–100 ppm in presence of copper chloride estimating the LOD of the dye as 7.77 ppm [y = (0.0007295)x + (0.21998 ± 0.00189)]; R^2^ = 0.99915]. The sensitivity investigation indicates that these three dye systems exhibit high sensitivity toward copper with a detection limit as low as 2.23 ppm.

### Selectivity Analysis

**Fig. 7:**
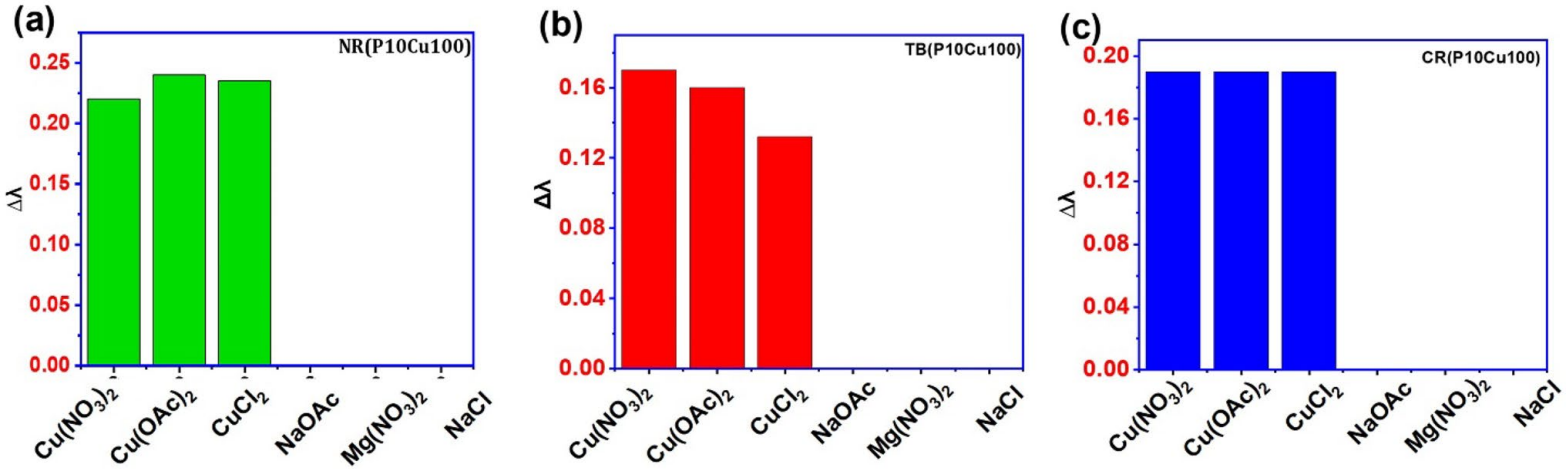
Selectivity analysis of different cations in different dye solutions: **a** Neutral red, **b** Thymol blue, and **c** Cresol red

To ensure the selectivity and specificity of the sensor, various test solutions representing other biomarkers in water such as sodium hydroxide, magnesium sulfate, potassium chloride, sodium chloride, and calcium carbonate were also added in the dye solutions. The test analyte was present at a concentration of 100 ppm, and a dye solution test was conducted at room temperature before UV–vis analysis. The findings demonstrated that all dye solutions exhibited a remarkable level of selectivity toward copper solutions. The dye’s selectivity for copper solutions was confirmed by measuring the relative change in the wavelength (∆λ) from UV–vis analysis estimated by the equation given below.

1 $$\Delta\uplambda =\frac{{\uplambda }_{\mathrm{x}}-{\uplambda }_{0}}{{\uplambda }_{0}}\times 100$$

where ${\uplambda }_{\mathrm{x}}$ stands for the specific wavelength of peak absorbance in the presence of the analyte and ${\uplambda }_{0}$ refers to the wavelength of maximum absorbance of the blank solution. The Δλ value is estimated at pH10 for Thymols blue, Cresol red, and Neutral red dyes. The data depicted in the Fig. 7 is showing the selectivity of the copper(II) solution at pH 10. The dye solutions detected the corresponding biomarkers only (Fig. 7). According to these findings, copper colorimetric detection was only marginally or barely impacted by other interfering chemicals. Therefore, the dye system is inert to copper detection and exhibits the potential for highly selective colorimetric sensor detection.

### Validation of Copper Ion Sensing Property of Dyes Coated Paper Sensor

Colorimetric detection is very easy for practical use and therefore it has been widely used in paper-based microfluidic sensors where a “yes/no” result is sufficient to give the results for any analysis. After the discovery of paper-based sensors by Martinez et al. [44] for the application in colorimetric detection of glucose and protein many developments have been achieved in this area. Nie et al. demonstrate the possibility of using an electrochemical paper sensing device for ethanol detection in food [45]. Paper-based sensors for copper ion detection are not very common. Nalin et al. reported a colorimetric sensor for selective detection of copper by using Ag nanoparticles [46]. In this work, Whatman No 1 filter paper was functionalized with the dye solutions by soaking it for 1 h followed by drying at room temperature overnight. To monitor the colour change initially 100 ppm concentration of copper acetate, chloride, and nitrate solution were poured into the functionalized filter papers and their changes were observed. A color change of red to orange in the case of Cresol red, and orange to red in the case of neutral red, while the color change from blue to yellow has been observed in the case of Thymol blue. Whatman No.1filter paper was functionalized by soaking it in dye solutions for 1 h and drying it at room temperature overnight. After which different concentration solutions ranging from 5–100 ppm were added to the functionalized filter paper, and changes in the color intensity were observed in different concentration variants.

**Fig. 8:**
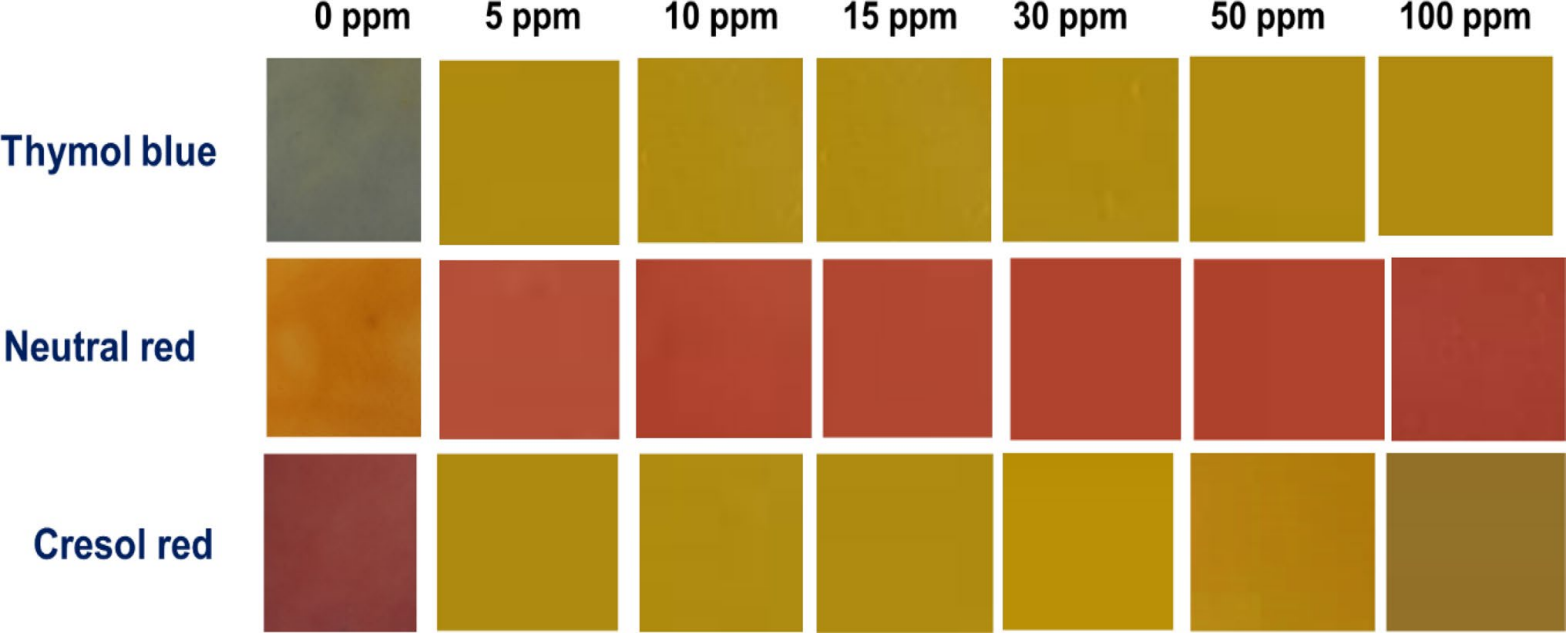
Color of the paper sensor at different copper (II) concentrations for long time exposure

Color maps of the sensor exposed to different concentrations of copper acetate were recorded. (Fig. 8). To freeze the hue a digital scanner was used immediately after the exposure. Images were captured in JPEG format and ImageJ software was used afterward to analyze the pictures. The JPEG pictures were examined after adjusting the settings (Hue adjustment section of the Threshold Color window) in ImageJ. Three similar zones were selected to verify the repeatability of the results. An increase in the colour intensity has been observed with the increase in the concentration of copper ion concentration. The blue colour of Thymol blue initially started to fade away at 5 ppm concentration and gradually changes to brown from orange on increase in the concentration from 10 to 100 ppm. In the case of Neutral red, the initial orange colour of the functionalized filter paper changed to red with the addition of 5 ppm copper solution. The intensity increases with an increase in the concentration of the copper solution. A similar trend has been observed in the case of Cresol red. A change in colour from red to yellow has been observed with the addition of 5 ppm of copper solution. This colour also intensified with the addition of consequently increased copper concentration. The variation in the change of colour is significantly visible for all the dye functionalized Whatman no1 paper. The LOD value obtained from this method for Cu(II) sensing has been compared with a few of the previously reported data in Table 3.

**Table 3:** Comparison of present LOD for Cu(II) detection with reported sensors

Sl. No	Sensors	Medium	LOD	Reference
1	Tetraphenylethene -Thiosemicarbazones sensor	Acetonitrile	15.74 ppm	[47, 48]
2	Based on azide-alkyne click chemistry	water	20 ppm	[40, 49]
3	Carboxymethyl gum karaya-capped gold nanoparticles	water	10 nM	[41, 50]
4	Papain-coated gold nanoparticles	water	200 nM	[42]
5	Pyrene and hydrazone	water	2.73 µM	[43]
6	Present work	water	2.23 ppm	-

This cost-effective paper base sensor can be utilized in copper ion detection in blood serum and urine. Humans generally excrete about 60 mcg/day of copper in the urine. A high concentration of copper is associated with the genetic disorder Wilson's disease. This may affect the normal liver function in the human body. By using cost-effective paper-based sensors it is possible to detect the concentration of copper in blood serum and urine.

**Fig. 9:**
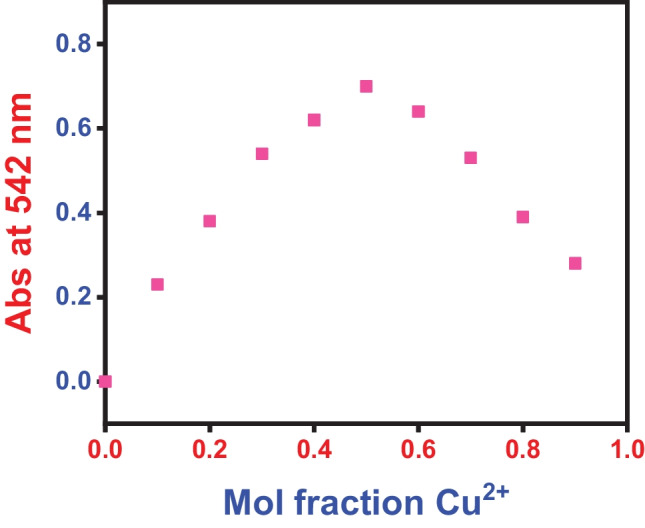
Job’s analysis of the Cresol red dye—Cu^2+^

The stoichiometry of Cresol red dye and copper (II) was validated by Job’s method; it is a widely used analytical technique to determine the stoichiometry of a binding event. This method keeps the total molar concentration of two binding components constant while varying the molar fraction of one binding component. In this study, the molar fraction of copper (II) was varied from 0 to 0.9 while keeping the sum of the initial concentration of dye and the copper(II) ion at 100 ppm. The absorbance for each molar fraction of copper (II) was recorded at 542 nm and was plotted against the molar fraction of the copper (II) ion, as shown in Fig. 9. The maximum absorbance was achieved at a molar fraction of 0.5, indicating a 1:1 stoichiometry of the SP1 and the copper (II) ion. This stoichiometry is consistent with previous colorimetric metal sensors [51-54] which often exhibited a 2:1 or 1:1 ligand − copper(II) stoichiometry.

## Conclusion

A paper-based sensor has been developed for selective colorimetric detection of copper ions using a dye-based sensing method, which can be easily detected by the naked eye. The rapid and highly selective detection of the copper ion with various counter ions like acetate, nitrate, and chloride makes it more versatile for using this paper-based sensor in the diverse application. The low detection limit of 2.23 ppm attained by this system is another advance associated with this developed method. Along with the high significantly low limit of detection, this probe is useful in the selective detection of copper in presence of commonly existing ions such as sodium, magnesium, potassium, and calcium. These simple and cost-effective paper-based sensors have great potential for the detection of copper ions in a biological samples such as blood serum and urine.

## Data Availability

The datasets generated during and/or analysed during the current study were included in the manuscripts butany additional data needed are will be available from the corresponding author on reasonable request.
